# Impact of postoperative choroidal detachment on trabeculectomy outcomes: a four-year comparative study

**DOI:** 10.1186/s12886-023-02860-1

**Published:** 2023-03-17

**Authors:** Maryam Yadgari, Mohammad Javad Ghanbarnia

**Affiliations:** 1 Ophthalmic Research Center, Research Institute for Ophthalmology and Vision Science, Shahid Beheshti University of Medical Sciences, Tehran, Iran; 2 Department of Ophthalmology, Imam Hossein Hospital, Shahid Beheshti University of Medical Sciences, Tehran, Iran; 3 Health Research Institute, Babol University of Medical Sciences, Babol, Iran

**Keywords:** Trabeculectomy, Choroidal detachment, Choroidal effusion, Glaucoma, Glaucoma surgery, Surgery outcomes, Intraocular pressure

## Abstract

**Background:**

The aim of this study was to compare trabeculectomy outcomes in patients with and without post-operative serous choroidal detachment (CD) and establish an association between CD and trabeculectomy outcomes.

**Methods:**

In this 4-year retrospective cohort study, medical records of glaucoma patients older than 18 who underwent primary trabeculectomy with Mitomycin-C between 2012 and 2020 were reviewed. Phakic eyes without history of any other intraocular surgery and with at least one year of follow-up were included in the study. Postoperative CD was defined as clinically visible CD developed within the first postoperative week. Cases were categorized into with and without CD and trabeculectomy outcomes were compared. Comparison was carried out using postoperative intraocular pressure (IOP), glaucoma medications and surgery success. Two levels of success were defined regardless of glaucoma medications; criteria A) 5 < IOP < 19 mmHg and criteria B) 5 < IOP < 16 mmHg. In addition to the defined IOP ranges, IOP reduction less than 20% from baseline and further glaucoma surgery were also counted as surgery failures.

**Results:**

Total of 183 patients including 153 without CD (mean age 58.73 ± 11.40 years, mean IOP 23.7 ± 6.63 mmHg) and 30 with CD (59.00 ± 12.59 years, mean IOP 22.2 ± 3.83 mmHg) entered the study. Post-trabeculectomy mean IOPs were significantly higher in the CD group at all follow-up visits at year 1 through 4 (14.70, and 14.82 mmHg vs. 11.03, and 12.59 mmHg; *p*-value < 0.05). Similarly mean number of glaucoma medications was higher in the CD group at all follow-up visits (*p*-value > 0.001). Based on success criteria A, cumulative probability of success for patients with CD wasn’t significantly different compared to those without CD at years 1 through 4 (80.0%, and 69.6% vs. 88.2%, and 74.1% respectively; *p*-value > 0.05, log-rank). However, based on success criteria B, patients with CD had significantly lower cumulative probability of success at years 1 through 4 (50.0% and 8.9% vs. 79.7% and 59.8%, *p*-value < 0.001).

**Conclusion:**

We established that early post-trabeculectomy serous choroidal detachment is associated with adverse surgery outcomes. Lower rate of surgery success and higher mean postoperative IOP and glaucoma medications were observed in patients with post-trabeculectomy choroidal detachment and this was more pronounced in patients who required more stringent IOP control (success definition 5 < IOP < 16 mmHg).

## Background

Trabeculectomy, since its first conception by Cairns [1], has been the incisional surgery of choice for management of glaucoma for most ophthalmologist in nearly six decades. Trabeculectomy allows for drainage of aqueous humour through a trans-scleral fistula, guarded by a sub-conjunctival filtering bleb [2]. It has been considered the gold standard of surgery for moderate to advanced glaucoma when strict Intraocular pressure (IOP) control is required and cases in which desired target IOP cannot be reached or progression of glaucoma cannot be halted despite the use of maximally tolerated IOP medications [3, 4]. In the past two decades, advancement of minimally invasive glaucoma surgery and development of drainage devices have caused a steady downward trend in the number of trabeculectomies performed by ophthalmologists. A study conducted on the USA health insurance data reported that trabeculectomy has decreased from 75,000 yearly cases in 1996 to 18,000 cases in 2012 [5]. In spite of this decline, trabeculectomy still remains a popular filtration surgery, as a separate study survey in 2016 reported that up to 60% of ophthalmologists prefer trabeculectomy for eyes with primary open angle glaucoma (POAG) without prior surgeries [6].

Over the years, modified surgical techniques and new medications have evolved trabeculectomy in order to reduce failure rate and minimize complications. Early trabeculectomies were associated with higher rates of bleb fibrosis and consequent bleb failure as a result of wound scarring [2, 3]. Hence, introduction of antifibrotic agents, namely Mitomycin-C (MMC) and 5-fluorouracil (5-FU), has significantly enhanced filtration bleb survival [2, 3]. This lead to widespread acceptance and popularity of trabeculectomy as an effective surgical modality in the 1990s, and by 2016 nearly 97% of primary trabeculectomies were performed with adjunctive MMC [6]. However, similar to other incisional surgeries, trabeculectomy is still susceptible to failure. Younger age, higher baseline IOP, diabetes, previous glaucoma surgery and intraocular surgery and post-surgery complications are among the identified risk factors for trabeculectomy failure [7–11]. Post-operative complications can influence the outcomes of trabeculectomy and they are a common occurrence; being reported in up to 50 percent of cases [8, 9]. Flat anterior chamber, wound leak, encapsulated bleb and choroidal detachment are among the most common early post-operative complications. Choroidal detachment is particularly noteworthy, since its occurrence following trabeculectomy is more frequent than other incisional surgeries [9, 12]. Furthermore, it can threaten vision by both directly impacting the retina and also potentially influencing the outcomes of trabeculectomy.

Choroidal detachment is a relatively common early post-trabeculectomy complication, occurring in 7.9 to 18.8 percent of cases [7–13]. The term choroidal detachment, used interchangeably with choroidal effusion, denotes accumulation of fluid in the suprachoroidal space. In normal eyes, suprachoroidal space is a potential space and its hydrostatic pressure is tightly balanced by an equilibrium between IOP and osmotic pressure from choriocapillaries. However, in trabeculectomy, an acute drop in IOP combined with inflammatory process causes a sudden shift in hydrostatic pressure. Consequently, serum transudates across choriocapillaries and accumulates in the suprachoroidal space [14, 15]. Risk factors for development of choroidal detachment following trabeculectomy include old age, large difference between pre and post-trabeculectomy IOPs, thicker cornea and hypotony [13, 16]. Hypotony and CD are highly correlated. Post-operative hypotony is a well-known etiology and risk factor for CD; furthermore, CD is a clinical presentation of hypotony in some cases [17, 18]. Studies concerning post-trabeculectomy hypotony have produced contradicting results as some demonstrated increased risk of bleb failure and sight threatening complications in eyes with post-operative hypotony [17], while others didn’t report any difference between eyes with and without hypotony [18]. However, the results of such studies aren’t necessarily applicable to eyes with CD, because not all hypotony cases lead to CD. Hence, investigating the impact of post-operative CD and establishing a connection between CD and surgery outcomes deems imperative.

Studies regarding CD and its effect on trabeculectomy outcomes have been limited and contradicting. Some earlier studies reported no association between early CD and long term visual and surgery outcomes [19, 20]. However, in a case control study, we previously indicated that patients who develop CD following trabeculectomy, have significantly higher long-term failure rates compared to patients that don’t develop CD [21]. In this Study, by implementing a larger sample size and a more stringent success definition, we aimed to improve limitations of our previous study and establish a more accurate association between CD and adverse surgery outcomes. Herein, we explored the four-year outcomes of trabeculectomy in patients who developed early post-operative serous choroidal detachment.

## Methods

In this retrospective cohort study, we reviewed medical records of patients that underwent primary trabeculectomy at Imam Hossein Hospital, Shahid Beheshti University of Medical Sciences, Tehran, between 2012 and 2020. All patients older than 18 years of age with primary open angle glaucoma (POAG), pseudoexfoliation glaucoma (PEX) or chronic angle closure glaucoma (CACG) with at least one year of available follow up data were included in this study. Primary trabeculectomy with MMC was performed on all of these patients by glaucoma specialists. In order to eliminate possible independent effects of lens status and intraocular surgery on the outcome of trabeculectomy, all aphakic and pseudophakic eyes, those with prior intraocular surgery and additional intraocular surgery during follow-up were excluded. In order to accurately determine the impact of CD and further minimize the possible confounding effects of other major post-operative complications on the outcome of trabeculectomy, major complications other than serous choroidal detachment were excluded from the study. Baseline characteristics of patients including age, glaucoma type, IOP, medications and visual acuity as well as post-operative complications and follow-up IOP and medications were extracted from the medical records. The study protocol adhered to the tenets of declaration of Helsinki. Ethics committee of Shahid Beheshti University of Medical Sciences, Tehran, Iran, approved this study.

### Surgical technique

All surgeries were performed by glaucoma specialists with at least 5 years of experience. Trabeculectomy with fornix-based conjunctival flap and trapezoidal 3 × 2 mm scleral flap at superior area was performed under general or local anesthesia. Then MMC (0.2 mg/ml) was applied by thin sponges under the scleral flap and conjunctiva for 2 min. After adequate irrigation, sclerectomy and peripheral iridectomy the scleral flap was closed by two 10–0 nylon releasable sutures and the conjunctiva was closed by 10–0 nylon sutures. Post-operatively, all patients were treated with chloramphenicol eye drop, 4 times a day for 1 week and betamethasone eye drop every 2 h for one week that was tapered off in 6–8 weeks. Post-operative evaluations, and further need for glaucoma medications were at the discretion of glaucoma specialists. The decision on removal and the timing of removal of releasable sutures were at the discretion of the glaucoma specialists and dependent on the post-operative course of IOP. In eyes with CD, releasable sutures were removed based on response to treatment and resolution of CD, as a result, they were removed in a more delayed fashion compared to eyes without CD. Releasable sutures were not removed for patients with persistent post-operative hypotony.

Based on post-operative examinations carried out by glaucoma specialists, patients were categorized into two groups; patients who developed early post-operative serous choroidal detachment (with CD) and those who didn’t (without CD). Post-operative CD was defined as clinically visible CD developed within one week following surgery and prior to removal of any releasable sutures. Diagnosis was made by glaucoma specialists through dilated fundus examination. B-scan ultrasound wasn’t routinely utilized, but rather reserved for suspicious cases or to rule out other conditions such as hemorrhagic CD or retinal detachment. Patients who developed CD were treated accordingly and almost all cases were resolved within one month of diagnosis. Management approach were at the discretion of the glaucoma specialist. These patients were treated with topical atropine and in unresponsive cases due to bleb overfiltration, topical corticosteroids were reduced or tapered more quickly. Majority of these cases were successfully treated with conservative medical therapy and surgical approach was reserved in vision-threatening or unresponsive cases.

For patients in both groups, baseline characteristics including age, glaucoma type, IOP, medications and visual acuity as well as post-operative complications and follow-up IOP and medications were extracted from the medical records. Post-operative IOP and number of IOP lowering medications were noted at months 1, 3, 6, 9, 12, 24, 36 and 48 following the surgery. Major complications and also the need for additional intraocular or glaucoma surgeries were also noted.

Trabeculectomy outcomes in groups with and without CD were compared based on three parameters; 1) post-operative IOP at follow-up visits 2) Requiring glaucoma medications following surgery 3) surgery success. Two levels of surgery success were defined based on post-operative IOP targets and regardless of glaucoma medications; criteria A) 5 < IOP < 19 mmHg and criteria B) 5 < IOP < 16 mmHg. For both criteria A and B, cases with IOP outside the defined target range or less than 20% reduction from baseline, during two consecutive visits at month three and later were considered surgery failures. The time point at which abnormal IOP was first observed, was then recorded as the time of failure. In addition, eyes with further glaucoma surgeries such as repeat trabeculectomy or tube shunt placement, and visual acuity reduction to no light perception were also considered surgery failure, regardless of the achieved IOP. Slit lamp procedures such as anterior chamber reformations were not considered as reoperation; hence, they were not counted as failure. Eyes that had not failed by the aforementioned criteria, but had required glaucoma medications to achieve target IOP were considered as qualified success. Eyes that had not failed by the defined criteria and also had not used IOP medications were counted as complete success. Overall surgery success consisted of the sum of all qualified and complete success cases, for the purposes of Kaplan–Meier survival analysis.

### Statistical analysis

Continuous variables are presented as means ± standard deviation. Categorical variables are presented as numbers and percentage. Normality of data distribution was evaluated using Shapiro–Wilk test of normality. Independent sample t-test or Chi-square test was performed to establish a difference between baseline characteristics of groups with and without CD. Independent sample t-test was utilized to compare mean IOP and number of IOP medications between the two groups at every follow-up visit. Number of complete success, qualified success and failed cases defined by either one of the two definitions of IOP target (criteria A and B) for four years of follow-up were compared using chi-square test. Kaplan–Meier survival analysis and log rank test were performed to evaluate probability of success of the groups with and without CD based on the two success definitions. In this analysis, failure (based on criteria A and B) was defined as the “event”. As a result, patients who dropped out of the study without any failure (i.e. lost to follow-up) and those who completed the full four years of study without failure were censored and this is shown with a plus ( +) sign on Kaplan–Meier graphs. *P*-values less than 0.05 were considered statistically significant. All Statistical analysis were conducted using SPSS version 26.0 (IBM, SPSS, Inc., Chicago, IL, USA).

## Results

After reviewing the medical records based on our inclusion and exclusion criteria from 2012 to 2020, 183 eyes of 150 consecutive patients entered the study including 153 (83.6%) eyes without CD and 30 (16.4%) cases with CD (Table 1). All 183 subjects completed at least one full year of follow-up, however some cases were lost to follow-up after the first year. In the second, third and fourth years of follow-up 125, 115 and 100 subjects remained in the study respectively. The overall mean age of all the study subjects was 58.78 ± 11.58 years. Mean baseline IOP was 23.45 ± 6.28 mmHg and mean number of glaucoma medications was 3.08 ± 0.86. Majority of the study subjects were male (*n* = 123, 67.21%) and POAG was the most common pre-operative diagnosis (*n* = 94, 51.37%). Comparison of the baseline characteristics of the two study groups are presented in Table 1. Patients with CD and without CD were mostly comparable in terms of their baseline characteristics (*P*-value 0.911, 0.302, 0.228, 0.600, 0.233 for age, VA, Sex, Glaucoma type, baseline IOP), except for the mean number of preoperative glaucoma medications which was higher in patients with CD (*p*-value 0.048).

**Table 1:** Baseline characteristics of patients entering the study in the case and control groups

**Parameter**		**Total**	**Group**		***P***
			**Without CD**	**With CD**	
**Number**		183	153(83.6%)	30 (16.4%)	-
**Age**	**Mean ± SD**	58.78 ± 11.58	58.73 ± 11.40	59.0 ± 12.59	0.911*
**Visual Acuity (LogMar)**	**Mean ± SD**	0.82 ± 0.62	0.79 ± 0.66	0.95 ± 0.32	0.302*
**Sex**	**Male**	123 (67.21%)	100 (65.36%)	23 (76.67%)	0.228**
	**Female**	60 (32.79%)	53 (34.64%)	7 (23.33%)	
**Type of glaucoma**	**POAG**	94 (51.37%)	78 (50.98%)	16 (53.33%)	0.600**
	**CACG**	49 (26.78%)	43 (28.1%)	6 (20%)	
	**PEX**	40 (21.86%)	32 (20.92%)	8 (26.67%)	
**Baseline IOP**	**Mean ± SD**	23.45 ± 6.28	23.7 ± 6.63	22.2 ± 3.83	0.233*
**Baseline anti-glaucoma medication**	**Mean ± SD**	3.08 ± 0.86	3.03 ± 0.88	3.37 ± 0.72	0.048*

*POAG* Primary open angle glaucoma, *CACG* Chronic angle closure glaucoma, *PEX* Pseudoexfoliation glaucoma

^*^Using independent sample t-test

^**^Using Chi-square test

Overall, 21(11.4%) cases (including 5 cases with CD and 16 without CD) underwent reoperation for glaucoma which consisted of 4 (2.19%) tube shunt surgeries and 17 (92.9%) repeat trabeculectomies. The five reoperation cases in those with CD consisted of 4 repeat trabeculectomies and 1 tube shunt placement. All cases of glaucoma reoperations were counted towards surgery failure. Persistent hypotony occurred in 8 patients all of whom were counted towards surgery failure. The remaining failure cases were as a result of inadequate IOP reduction based on criteria A and B. Almost all CD cases were conservatively treated and resolved within one month of diagnosis. One patient underwent choroidal drainage. For one patient anterior chamber reformation was performed and one other patient underwent conjunctival suturing for wound leak. Additional scleral flap suture placement was performed for four patients to restrict flow.

**Fig. 1:**
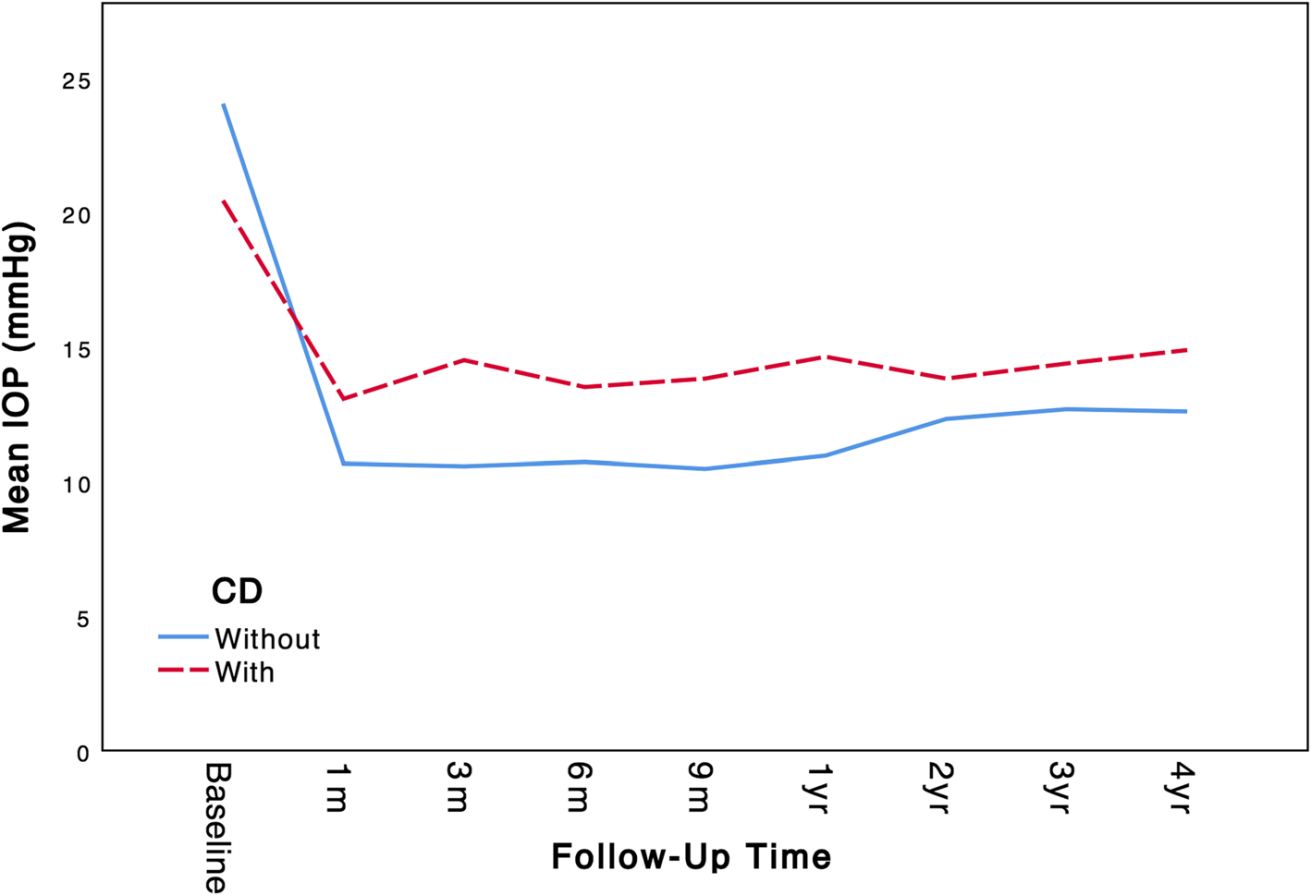
Graph showing the trend of mean IOP (mmHg) in groups with and without CD at baseline and follow-up visits. Cases which were missing the complete four-year follow-up data aren’t included in this graph

**Fig. 2:**
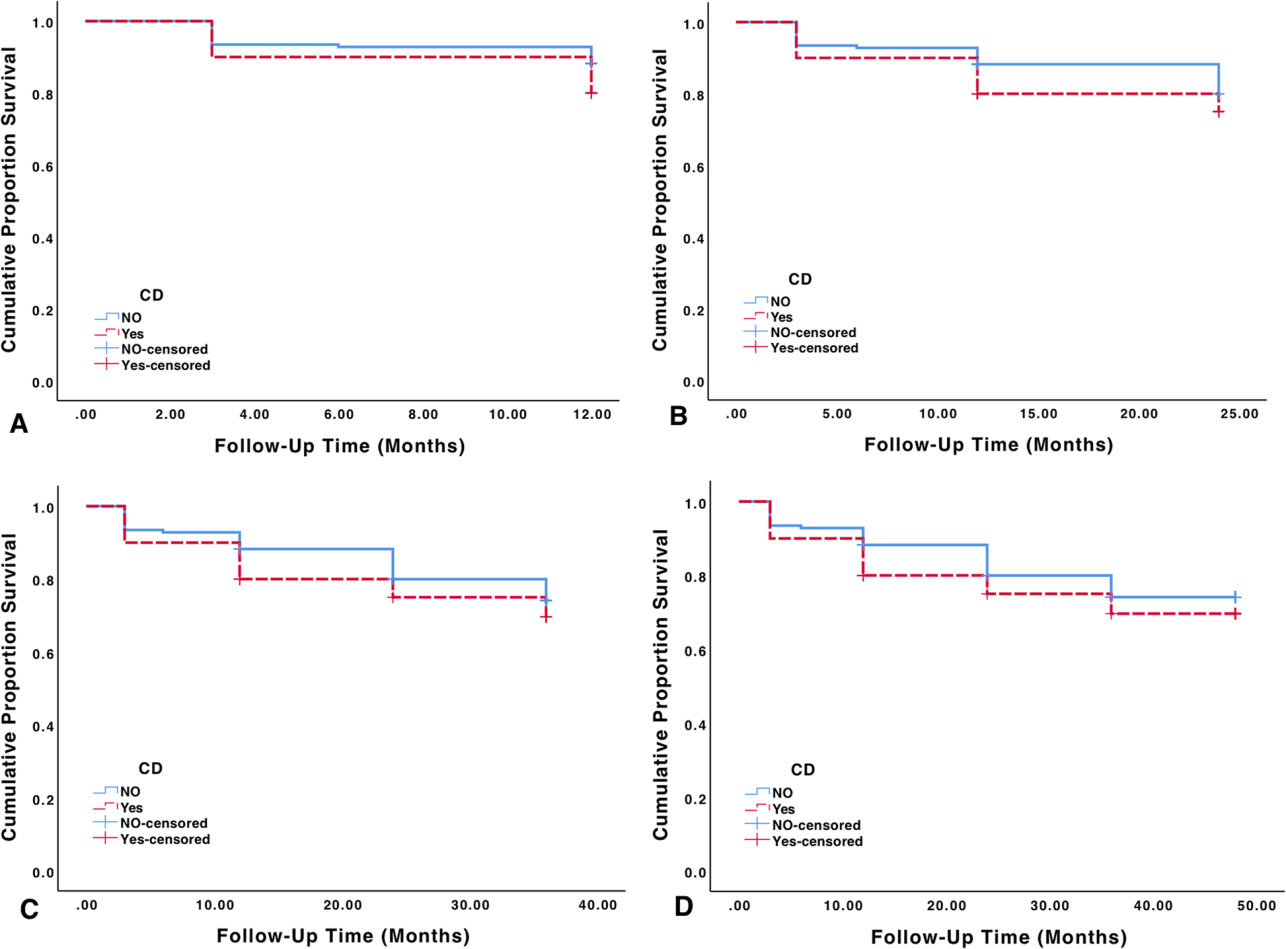
Kaplan–Meier cumulative probability of success plots showing the proportion of successful surgery outcomes at follow-up times 12 months (**A**), 24 months (**B**), 36 months (**C**) and 48 months (**D**). Successful outcome was defined using the 5 < IOP < 19 mmHg criteria. Patients with IOP reduction less than 20% from baseline, those with glaucoma reoperation and loss of light perception were still defined as failure

Changes in mean IOP and mean number of glaucoma medications in groups with and without CD at every follow-up visit are presented in Table 2. Even though the CD group started with lower mean baseline IOP compared to the group without CD (22.2 ± 3.83 vs. 23.7 ± 6.63 mmHg), the group with CD ended up with higher mean IOP at the end of four years (14.82 ± 3.00 vs. 12.59 ± 3.19 mmHg). At every follow-up visit, mean IOP and mean number of glaucoma medications significantly decreased compared with baseline (*p*-value < 0.001, using paired sample t-test). Furthermore, mean IOP was significantly higher in the group with CD at all follow-up visits year 1, 2, 3, and 4 (*p*-value < 0.001, 0.009, 0.035 and 0.009 respectively) as indicated in Table 2. Mean number of glaucoma medications was also significantly higher in the CD group at all follow-up visits (*p*-value < 0.001, 0.001, 0.009, < 0.001 for years 1 through4 respectively). The trend of variations in mean IOP over time in both groups is demonstrated in Fig. 1. Unlike the comparison established in Table 2, the IOP trend presented in Fig. 2 only includes cases that completed the full four-year follow-up (100 cases). Hence, mean IOP at every follow up, except the fourth year, were slightly different compared to values of Table 2.

**Table 2:** Comparison of Mean IOP and medcations of patients with and without CD at baseline and follow-up visits

Follow-up Time	N	Total	Group		P^*^
			Without CD	With CD	
**Baseline**	183				
IOP (mmHg)		23.45 ± 6.28	23.7 ± 6.63	22.2 ± 3.83	0.233
Medications		3.08 ± 0.86	3.03 ± 0.88	3.37 ± 0.72	**0.048**
**Month 1**	183				
IOP		10.88 ± 3.53	10.47 ± 3.28	12.97 ± 4.04	**< 0.001**
Δ IOP^a^		12.57 ± 7.36	13.23 ± 7.45	9.23 ± 5.98	**0.006**
Medications		0.12 ± 0.52	0.08 ± 0.41	0.3 ± 0.88	**0.038**
Δ Medications^b^		2.96 ± 1	2.94 ± 0.98	3.07 ± 1.08	0.530
**Month 3**	183				
IOP		11.08 ± 3.48	10.57 ± 3.2	13.7 ± 3.69	**< 0.001**
Δ IOP		12.37 ± 7.41	13.13 ± 7.44	8.5 ± 6.03	**0.002**
Medications		0.3 ± 0.71	0.19 ± 0.55	0.87 ± 1.07	**< 0.001**
Δ Medications		2.78 ± 1.03	2.84 ± 1	2.5 ± 1.14	**0.100**
**Month 6**	183				
IOP		11.25 ± 3.16	10.93 ± 3.05	12.87 ± 3.22	**0.002**
Δ IOP		12.2 ± 7.31	12.76 ± 7.45	9.33 ± 5.86	**0.018**
Medications		0.4 ± 0.81	0.32 ± 0.73	0.83 ± 1.05	**0.001**
Δ Medications		2.68 ± 1.07	2.71 ± 1.08	2.53 ± 1.07	0.423
**Month 9**	183				
IOP		11.33 ± 3.24	10.92 ± 3.28	13.43 ± 2.1	**< 0.001**
Δ IOP		12.12 ± 6.98	12.78 ± 7.18	8.77 ± 4.62	**0.003**
Medications		0.62 ± 1.02	0.51 ± 0.94	1.20 ± 1.24	**0.001**
Δ Medications		2.46 ± 1.19	2.52 ± 1.2	2.17 ± 1.15	0.143
**Year 1**	183				
IOP		11.63 ± 3.22	11.03 ± 2.88	14.70 ± 3.16	**< 0.001**
Δ IOP		11.83 ± 7.12	12.67 ± 7.15	7.50 ± 5.18	**< 0.001**
Medications		0.92 ± 1.17	0.71 ± 1.04	1.97 ± 1.25	**< 0.001**
Δ Medications		2.16 ± 1.29	2.31 ± 1.25	1.4 ± 1.25	**< 0.001**
**Year 2**	125				
IOP		13.07 ± 4.75	12.56 ± 4.36	15.45 ± 5.79	**0.009**
Δ IOP		10.12 ± 7.77	11.09 ± 7.83	5.59 ± 5.69	**0.002**
Medications		1.36 ± 1.32	1.17 ± 1.28	2.23 ± 1.19	**0.001**
Δ Medications		1.67 ± 1.38	1.83 ± 1.32	0.95 ± 1.43	**0.007**
**Year 3**	115				
IOP		13.16 ± 3.55	12.86 ± 3.56	14.78 ± 3.17	**0.035**
Δ IOP		10.02 ± 7.16	10.71 ± 7.46	6.28 ± 3.52	**0.015**
Medications		1.48 ± 1.33	1.34 ± 1.33	2.22 ± 1.11	**0.009**
Δ Medications		1.51 ± 1.37	1.65 ± 1.31	0.78 ± 1.48	**0.012**
**Year 4**	100				
IOP		12.97 ± 3.25	12.59 ± 3.19	14.82 ± 3.00	**0.009**
Δ IOP		10.47 ± 7.76	11.45 ± 7.96	5.71 ± 4.34	**0.005**
Medications		1.56 ± 1.31	1.30 ± 1.26	2.82 ± 0.73	**< 0.001**
Δ Medications		1.38 ± 1.29	1.61 ± 1.23	0.24 ± 0.9	**< 0.001**

Mean IOPs are presented as mmHg and number of medications are a number between one and four

^*^Using independent sample t-test

^a^Δ IOP represents the change in mean IOP from baseline (mmHg)

^b^ΔMedications represents the change in number of IOP medications used from baseline (number between 1 and 4)

Four-year Outcomes of trabeculectomy stratified by cumulative failure, complete success and qualified success are presented in Table 3. The first row presents surgery outcomes based on criteria B (5 < IOP < 16 mmHg). As per definition, patients with IOP reduction less than 20% from baseline and those with glaucoma reoperations were also defined as failure. In study subjects without CD, 31 (20.3%) of cases failed at year one and this number increased to 53 (48.6%) by year four (Table 3). Within the group with CD, 15 cases (50.0%) failed at year one and by year four, 20 cases (95.2%) who still remained in the study had failed. The number of failed subjects in the group with CD was significantly higher compared with cases without CD at every year following trabeculectomy (*p*-value < 0.001 using chi-square, and largest standardized residual for failure in the CD group).

**Table 3:** Breakdown of cumulative failure, complete success and qualified success cases stratified by presence or absence of postoperative CD at follow-up visits years one through four

	Year	Total N	Total			Without Choroidal Detachment			With Choroidal Detachment			P*
			Failure (%)	Complete Success	Qualified Success	Failure	Complete Success	Qualified Success	Failure	Complete Success	Qualified Success	
5 < IOP < 16	Y1	183	46 (25.1)	91 (49.7)	46 (25.1)	31 (20.3)	86 (56.2)	36 (23.5)	15 (50.0)	5 (16.7)	10 (33.3)	< 0.001
	Y2	141^a^ (125 + 16)	58 (41.1)	37 (26.2)	46 (32.6)	41 (34.5)	36 (30.3)	42 (35.3)	17 (77.3)	1 (4.5)	4 (18.2)	< 0.001
	Y3	137^a^ (115 + 22)	68 (49.6)	29 (21.2)	40 (29.2)	48 (41.4)	29 (25.0)	39 (33.6)	20 (95.2)	0 (0.0)	1 (4.8)	< 0.001
	Y4	130^a^ (100 + 30)	73 (56.2)	25 (19.2)	32 (24.6)	53 (48.6)	25 (22.9)	31 (28.4)	20 (95.2)	0 (0.0)	1 (4.8)	< 0.001
5 < IOP < 19	Y1	183	24 (13.1)	93 (50.8)	66 (36.1)	18 (11.8)	88 (57.5)	47 (30.7)	6 (20.0)	5 (16.7)	19 (63.3)	< 0.001
	Y2	136^a^ (125 + 11)	34 (25.0)	38 (27.9)	64 (47.1)	27 (23.7)	37 (32.5)	50 (43.9)	7 (31.8)	1 (4.5)	14 (63.6)	0.028
	Y3	130^a^ (115 + 15)	41 (31.5)	29 (22.3)	60 (46.2)	33 (30.3)	29 (26.6)	47 (43.1)	8 (38.1)	0 (0.0)	13 (61.9)	0.026
	Y4	119^a^ (100 + 19)	41 (34.5)	25 (21.0)	53 (44.5)	33 (33.3)	25 (25.3)	41 (41.4)	8 (40.0)	0 (0.0)	12 (60.0)	0.038

^*^Based on Chi-Square Tests

^a^Number of participants who remained in the study, plus those who were lost to follow up but had already failed based on previous follow-up visits

The second row of Table 3 breaks down the cumulative failure, complete success and qualified success cases based on criteria A (5 < IOP < 19 mmHg) at years one through four. Similarly, cases with IOP reduction less than 20% from baseline and those with glaucoma reoperation were still defined as failure. The number of complete success subjects in the group with CD was significantly lower compared with the group without CD at all follow-up visits (*p*-value < 0.001, 0.028, 0.026 and 0.038 in years one through four using chi-square test, and the largest standardized residual value for complete success in the CD group).

The primary outcome measure was to evaluate probability of success for individuals with and without CD. For this purpose, Kaplan–Meier survival analysis was performed. Figure 2 demonstrates four probability of success plots (success criteria A 5 < IOP < 19 mmHg) each depicting the cumulative probability of successful outcomes by post-operative follow-up times; year 1 (Fig. 2A), year 2 (Fig. 2B), year 3 (Fig. 2C) and year 4 (Fig. 2D). Patients with IOP reduction less than 20% from baseline, those with further glaucoma reoperation and loss of VA to no light perception were also counted as failure. Estimated mean time to failure for years 1, 2, 3 and 4 following trabeculectomy in the group with CD was 11.10, 20.70, 29.70 and 38.06 months respectively (Fig. 2). Corresponding values for the group without CD are 11.37, 21.96, 31.56 and 40.45 months respectively. The difference in time to failure between the two groups wasn’t statistically significant at any follow-up visit (*p*-value 0.228, 0.411, 0.458 and 0.458 for years 1 through 4 respectively, using log rank test). Cumulative probability of success for patients without CD was 88.2, 80.0, 74.1 and 74.1% for years 1 to 4 respectively while corresponding values for patients with CD were 80.0, 75.0, 69.6 and 69.6% for years 1 to 4 respectively.

**Fig. 3:**
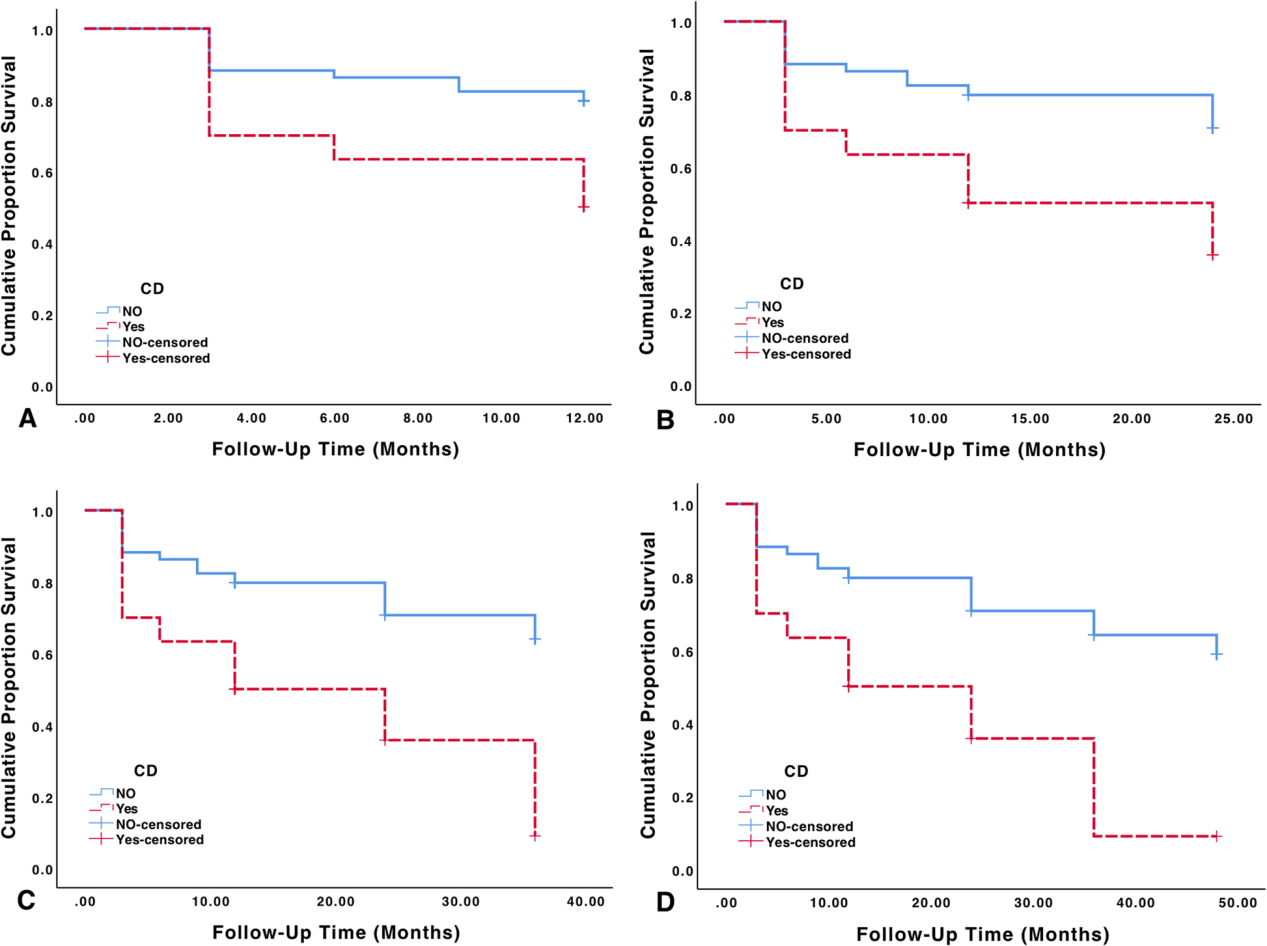
Kaplan–Meier cumulative probability of success plots showing the proportion of successful surgery outcomes at follow-up times 12 months (**A**), 24 months (**B**), 36 months (**C**) and 48 months (**D**). Successful outcome was defined using the 5 < IOP < 16 mmHg criteria. Patients with IOP reduction less than 20% from baseline and those with glaucoma reoperation and loss of light perception were still defined as failure

Kaplan–Meier cumulative probability of success plots based on the stricter success criteria B (5 < IOP < 16 mmHg) are presented in Fig. 3. Cumulative proportion of successful surgery outcomes at years 1, 2, 3, and 4 are presented in Fig. 3A, B, C and D respectively. Estimated mean time to failure for the group with CD at years 1, 2, 3 and 4 was 8.90, 14.90, 19.19 and 20.26 months respectively. For the group without CD, corresponding values were 10.71, 20.28, 28.76 and 36.44 months. Estimated time to failure was significantly lower in the group with CD compared with cases without CD at every yearly follow-up visit (*p*-value < 0.001 at years 1 through 4, using log rank test). This is in contrast to the results obtained by using criteria A which produced no significant difference between the two groups at any follow-up visits. Furthermore, cases without CD had cumulative probability of success of 79.7, 70.7, 64.1 and 58.8% at years 1, 2, 3 and 4 following trabeculectomy, while the corresponding values for those with CD was 50.0, 35.7, 8.9 and 8.9%.

## Discussion

In this cohort study, we established that post-operative serous choroidal detachment is associated with adverse trabeculectomy outcomes. Both mean IOP and mean number of glaucoma medications were significantly higher in cases with CD after four years of follow-up compared with those without CD (14.82 mmHg and 2.82 vs 12.59 mmHg and 1.30; *p*-value 0.009 and < 0.001 respectively). In this study, the overall cumulative probability of success for all subjects combined, both with and without CD, at three years of follow-up was 73.4% based on success criteria A (5 < IOP < 19 mmHg). This is comparable to studies with similar success definitions at three years which reported 72.0, 69.0 and 77.0% probability [9, 22, 23]. Their overall surgery success at three years using criteria B was 51.0, 61.3 and 56.0% respectively [9, 22, 23] which are also comparable to our combined probability of success of 57.1%. Interestingly, in our study, the group with CD alone achieved cumulative probability of success of only 8.9% using the same criteria at three years which is considerably lower. This hints at the possible adverse effect of CD on trabeculectomy outcomes and highlights the need for such comparisons to be made.

Serous choroidal detachment is highly correlated with post-operative hypotony. Acute drop in IOP following glaucoma surgeries causes transudation of serous fluid through choroidal capillaries and into the suprachoroidal space; hence, post-trabeculectomy hypotony is a contributor to CD [13–16]. Some studies have already explored the effect of post-trabeculectomy hypotony on surgery outcomes [17, 18]. However, the results of these studies aren’t necessarily applicable to patients with CD mainly because of differences in study methods. It’s also worth noting that CD develops only in some hypotony cases. Benson et al. [17] indicated that early post-operative hypotony is associated with significantly higher rate of trabeculectomy failure after 5 years of follow-up [17]. They defined hypotony as IOP < 8 mmHg or IOP < 10 mmHg with clinical signs such as CD. However, they didn’t specify how many of the study subjects involved cases with CD. The time intervals between surgery and IOP measurements is also unclear, as early transient post-surgery hypotony doesn’t necessarily lead to CD. Furthermore, the authors excluded trabeculectomies with mitomycin-C from the study and it is unclear how the results would have changed if they were included. In a separate 6-year case–control study, Tseng et al. studied patients who developed post-operative hypotony at three months or later following trabeculectomy [18]. In contrast to Benson et al. they indicated that there was no significant association between hypotony and probability of trabeculectomy failure or vision loss after 6 years [18]. They further concluded that, although clinically important, numeric hypotony isn’t necessarily accompanied by adverse surgery outcomes [18]. Again in this study there isn’t enough CD cases, similar to Benson et al.’s study, in order for accurate conclusions to be drawn.

Studies that evaluated the effect of CD on trabeculectomy outcomes are limited and contradicting. Stewart et al. conducted a case control study with a one-year follow-up involving 18 patients who developed early post-operative serous CD and 18 control patients [20]. Each group consisted of 8 phakic and 10 pseudophakic eyes. In contrast to our study, they reported that at one year following trabeculectomy, no significant difference in mean IOP between the two groups was observed (15.2 mmHg vs. 15.9 mmHg). Moreover, the number of glaucoma medications used by year one didn’t differ significantly between the two groups. They further concluded that severity, duration and time of onset of CD was not a determinant of final IOP [20]. Older age of participants (67.5 vs 59.0 years in our study) and prior intraocular surgery are among the factors responsible for contradicting results. Some studies have already established that both factors can independently influence surgery success [7, 23]. Moreover, low number of phakic eyes in each group (*n* = 8) challenges accurate statistical analysis. Number of cases that required reoperation is also unclear in Stewart et al.’s study. Perhaps if they had defined a surgery success criteria and performed survival analysis like in this study, they would have achieved different results. In a separate study, Popovic et al. examined post-operative outcomes in patients with early CD following trabeculectomy [24]. In their study, 17 cases including 10 CD patients were followed for mean time of 19.4 months. They concluded that IOP was not significantly different between the two groups (15.4 mmHg vs 15.1 mmHg in CD and without CD groups respectively) [24]. In contrast to our study trabeculectomies were performed using the limbal based approach and no antimetabolite such as Mitomycin-C was used. It is also unclear as to how the two groups differed in terms of glaucoma medications at every follow-up visit as this could significantly alter the conclusion of the study. Mean age of participants in Popovic et al.’s study was considerably higher compared to our study (73 ± 8.3 years vs. 58.78 ± 11.58) and three out of ten CD (30%) cases weren’t clinically evident and diagnosis was based on ultrasound as opposed to ophthalmoscopy. Small sample size (10 CD) hinders accurate analysis, since 30% of CD cases weren’t clinically significant, in contrast to our study that all CD cases were clinically significant.

In a recent study, Rao et al. reported one-year clinical outcomes of eyes with post primary trabeculectomy CD [25]. They included a total of 45 patients with primary open angle glaucoma (POAG) and primary angle closure glaucoma (PACG) with mean age of 62.86 ± 8.8 years. All patients included in the study had developed CD and they were not compared to their counterparts without CD. After one year of follow-up cumulative probability of overall success was 79.3% [25], which is comparable to 80.0% cumulative probability of success for cases with CD in our study using criteria A. However, it should be noted that Rao et al. implemented a higher IOP target as surgery failure compared to this study (IOP > 21 mmHg vs IOP > 19 mmHg). Several major design differences hinder accurate comparison of the two studies. Our study participants consisted of 20.8% patients with pseudoexfoliation glaucoma (PEX), whereas Rao et al. didn’t include PEX in their study. The majority of participants in Rao et al.’s study underwent phaco trabeculectomy, as opposed to trabeculectomy in our study. In contrast to our study, Rao et al. didn’t discriminate against history of previous intraocular surgery and 12 out of 45 eyes underwent additional cataract surgery during follow-up. Rao et al. didn’t include eyes without CD in their study; hence, accurate comparison cannot be made.

Altan et al. conducted a study that was most similar in design to our current study [19]. By including 225 patients without CD and 28 patients with CD in the study, they aimed to assess prognostic importance of CD on adverse post-operative outcomes. They reported that patients who developed CD following trabeculectomy had significantly lower best corrected visual acuity (BCVA) scores and also significantly higher cup to disc ratio; possibly suggesting that those who developed CD were in more advanced stages of glaucoma [19]. They also reported that by the end of the 2-year follow-up the CD group used significantly more glaucoma medications compared with the group without CD (mean 1.16 vs 0.68 respectively) which is consistent with our findings in this study. In terms of surgical success, Altan et al. reported no significant difference between the two groups at 2 years of follow-up [19]. Comparably, in this study we demonstrated that at 2 years, patients with and without CD didn’t have significantly different time to failure using criteria A (20.70 vs 21.96 months; *p*-value 0.411 log rank test). However, time to failure was significantly lower in CD cases compared to those without CD using criteria B which implemented a more stringent target IOP (14.90 vs 20.28 months; *p*-value < 0.001). Altan et al.’s criteria for inadequate IOP decrease (IOP ≥ 18 mmHg) was closer but not quite similar to our criteria A (5 < IOP < 19 mmHg). Furthermore, in their failure definition, they didn’t include less than 20% reduction from baseline IOP, need for additional glaucoma surgeries and persistent hypotony all of which could potentially alter the results of the study. Survival analysis was also not performed in their study. Although there are some similarities between the two studies, definitive conclusion cannot be drawn due to design differences.

In a case–control study, we previously demonstrated that CD was associated with adverse trabeculectomy outcomes in the long run [21]. In the current study we also achieved similar results; though, we aimed to improve our study design and reduce short-comings of our previous study. As opposed to the previous study, we implemented a cohort-based approach with larger sample population. Furthermore, cases with major post-surgery complications other than CD and those who underwent intraocular surgery (e.g. cataract extraction) during the follow-up period were excluded. Combined with a more thorough statistical analysis, this study was better equipped to isolate the effect of CD on surgery outcomes more accurately. Nowadays, with improvements in glaucoma medications, laser glaucoma surgeries and minimally invasive glaucoma surgeries, trabeculectomy is more commonly performed in moderate to severe glaucoma cases. Initial trabeculectomy is more beneficial in patients with more advanced visual field loss at presentation [26]. Furthermore, it has been accepted that patients with advanced glaucoma require lower IOP targets compared with mild cases [27, 28]. Therefore, in this study we set the upper limit of target IOP at 16 mmHg (criteria B; 5 < IOP < 16 mmHg) in order to simulate the effect of CD on surgery outcomes in patients who require stringent IOP control (E.g. moderate to advanced glaucoma cases). When inadequate IOP reduction was defined based on criteria A (5 < IOP < 19 mmHg), estimated mean duration of surgery success wasn’t significantly different between cases with and without CD at all follow-up visits as shown in Fig. 2 (*p*-value 0.228, 0.411, 0.458 and 0.458, log rank test; years 1, 2, 3and 4 respectively). In contrast, estimated mean duration of success was significantly longer in cases without CD at all follow-up visits when the more stringent criteria B (5 < IOP < 16) was implemented (Fig. 3). This is similar to our previous study, where the 5-year surgery success duration was significantly longer in cases without CD [21]. Moreover, both studies indicated that mean post-operative IOP and glaucoma medications are significantly higher in cases with post-surgery CD at both short and long term. As a result, consistent with our previous study [21], we conclude that development of early choroidal detachment following trabeculectomy has adverse effects on the outcomes of surgery. Furthermore, adverse outcomes are more pronounced in patients who require a more stringent IOP target.

Several factors can possibly contribute to the increased probability of surgery failure in patients with CD. Even though the exact mechanisms by which adverse outcomes occur in these patients are still unclear, majority of explanations point towards management approach of CD following surgery, such as less aggressive bleb manipulation and reduction of topical corticosteroids in some cases as needed. Conservative measures for treatment of CD in cases with bleb overfiltration sometimes involves reduction of topical corticosteroids, which in turn contributes to higher rate of bleb fibrosis and consequent bleb failure [29]. De Barros et al. reported that in cases with concurrent anterior chamber shallowing, medicinal therapy alone is more likely to be associated with higher IOP compared to AC reformation with viscoelastic [30]. While some studies suggested that combined AC reformation and CD drainage may be accompanied by more desirable surgery outcomes [30], other studies noted that mechanical pressure applied to the bleb as a result of AC reformation or CD drainage may lead to bleb fibrosis [31]. Further studies are required to more accurately identify and validate the mechanisms by which CD causes trabeculectomy failure.

There are some limitations to this study. First, retrospective design of this study hindered data collection and availability. Some patients’ records had missing values and data, consequently, some variables such as glaucoma stage and duration couldn’t be included in the study. Moreover, not all patient records contained the full four years of follow data, as a result, 183 patients were included in the first year which decreased to 100 patients by the fourth year. Second, although exclusion of aphakic and pseudophakic eyes and eyes with prior intraocular surgery can be viewed as a study strength, it can also be considered a limitation; since the study results cannot necessarily be applied to patients with said conditions.

In conclusion, we established that post-operative serous choroidal detachment is associated with adverse trabeculectomy outcomes. Higher rate of surgery failure, mean post-operative IOP and glaucoma medications were observed in patients with CD. Those with post-operative CD were less likely to achieve stringent IOP targets in four years of follow-up. Post-operative CD should be considered as a risk factor for trabeculectomy failure and it requires deliberate pre-operative risk assessment.

## Data Availability

The datasets used and/or analyzed during the current study are available from the corresponding author on reasonable request.
